# Drinking natural water unchangeably is associated with reduced all-cause mortality in elderly people: A longitudinal prospective study from China

**DOI:** 10.3389/fpubh.2022.981782

**Published:** 2022-08-22

**Authors:** Lu Liu, Yi Zheng, Haiyan Ruan, Liying Li, Liming Zhao, Muxin Zhang, Linjia Duan, Sen He

**Affiliations:** ^1^Department of Cardiology, West China Hospital of Sichuan University, Chengdu, China; ^2^Department of Cardiology, Hospital of Traditional Chinese Medicine of Shuangliu District, Chengdu, China; ^3^Department of Cardiovascular Medicine, Hospital of Chengdu Office of People's Government of Tibetan Autonomous Region, Chengdu, China; ^4^Department of Cardiology, First People's Hospital of Longquanyi District, Chengdu, China

**Keywords:** tap water, natural water, drinking water sources, mortality, elderly people

## Abstract

**Objective:**

Because of rapid economic growth and followed urban expansion in China, many people drinking natural water had to change their water sources to tap water. We aimed to test the unknown association that whether continued use of natural water for drinking is different from switching to tap water in all-cause mortality risks in elderly people.

**Methods:**

In total, based on Chinese Longitudinal Healthy Longevity Survey, 26,688 elderly participants drinking natural water from childhood to young-old were included in the final analyses. Associations between whether changing drinking water sources or not and all-cause mortality risk were then estimated by Cox regression models with the use of multiple propensity score methods, and the primary analysis used propensity score matching, with other propensity score methods confirming the robustness of the results.

**Results:**

Baseline characteristics were fairly well balanced by the three post-randomization methods. During a median follow-up period of 3.00 (IQR: 1.52, 5.73) years, 21,379 deaths were recorded. The primary analysis showed people using natural water unchangeably was associated with a lower risk of all-cause mortality than those switching to tap water in later life (HR: 0.94, 95% CI: 0.91–0.97, *p* < 0.001). Other propensity score methods, as well as Cox regression analysis without using propensity score methods, showed similar results.

**Conclusions:**

Among elderly people depending on natural water for drinking from their childhood to young-old in China, continued use of natural water was associated with a lower all-cause mortality risk than conversion to tap water later. Further studies in different countries and populations are needed to verify our conclusions.

## Introduction

In countries with an advanced economy, science and technology, the choice of water sources such as tap water, bottled water, natural mineral water, and others is a privilege for residents (1). Besides the considerations of accessibility, price difference, tastes, odors, and environmental protection, drinking water safety is increasingly becoming a major concern (2–5). Major contamination in drinking water often comes from heavy metals, harmful substances, and pathogens (2).

People often associate tap water with chemicals, chlorine, sediments, etc., (5, 6), leading to increased demand and sales for bottled water and mineral water to some extent (1, 4, 5, 7). Over the previous decades, because of sometimes reports of water supply network contamination (8), tap water was occasionally a controversial topic (1). When it comes to natural waters, take mineral water for an example, it has different characteristics from tap water and originates from protected underground water sources, and is subjected to different safety regulations as well (9). However, despite the microbiological wholesomeness of mineral water (10), very recent reports determined microplastic particles in it (11), arousing public attention.

Rapid economic growth and its huge population have accelerated the urban expansion in China in the past decades. People flooded into metropolises and used tap water in most daily life. At the same time, there was still a considerably large amount of population staying in the countryside and obtaining water directly from rivers, lakes, wells, pools, springs, etc., (12). As far as we know, recent studies mainly concentrated on the associations between components in drinking water and health problems (13–15), the data on the impact of different drinking water sources on long-term survival or mortality is limited. Thus, we aimed to explore whether consistently drinking natural water makes difference from converting to tap water on all-cause mortality in elderly people from a longitudinal prospective study in China.

## Materials and methods

### Study participants

Data were obtained from the Chinese Longitudinal Healthy Longevity Survey (CLHLS), a nationwide, ongoing, prospective cohort study of community-dwelling Chinese elderly people. The CLHLS aimed to examine the social, behavioral, biological, and environmental determinants of healthy human longevity and oldest-old mortality.Briefly, the survey adopted a targeted random-sample design to ensure representativeness, through interviews with approximately equal numbers of male and female non-agenarians, octogenarians, and young-old (aged 65–79 years) living near to the centenarians (in the same village or street, if available, or in the same sampled county or city) and was conducted in a half of the counties and cities in 23 provinces, covering about 85.0% of the total population of China. The CLHLS began in 1998, with subsequent follow-ups in 2000, 2002, 2005, 2008, 2011, 2014, and 2018. To reduce the attrition due to death and loss to follow-up, new participants are enrolled during the following waves from 1998.These waves were administered in participants' homes by trained interviewers with a structured questionnaire. Other details concerning the objectives, design and methods of the CLHLS can be found elsewhere (16, 17). The CLHLS complied with the principles of the Declaration of Helsinki (18), and was approved by the Research Ethics Committee of Peking University (IRB00001052-13074). All participants or their proxy respondents provided written informed consent.

**Figure 1:**
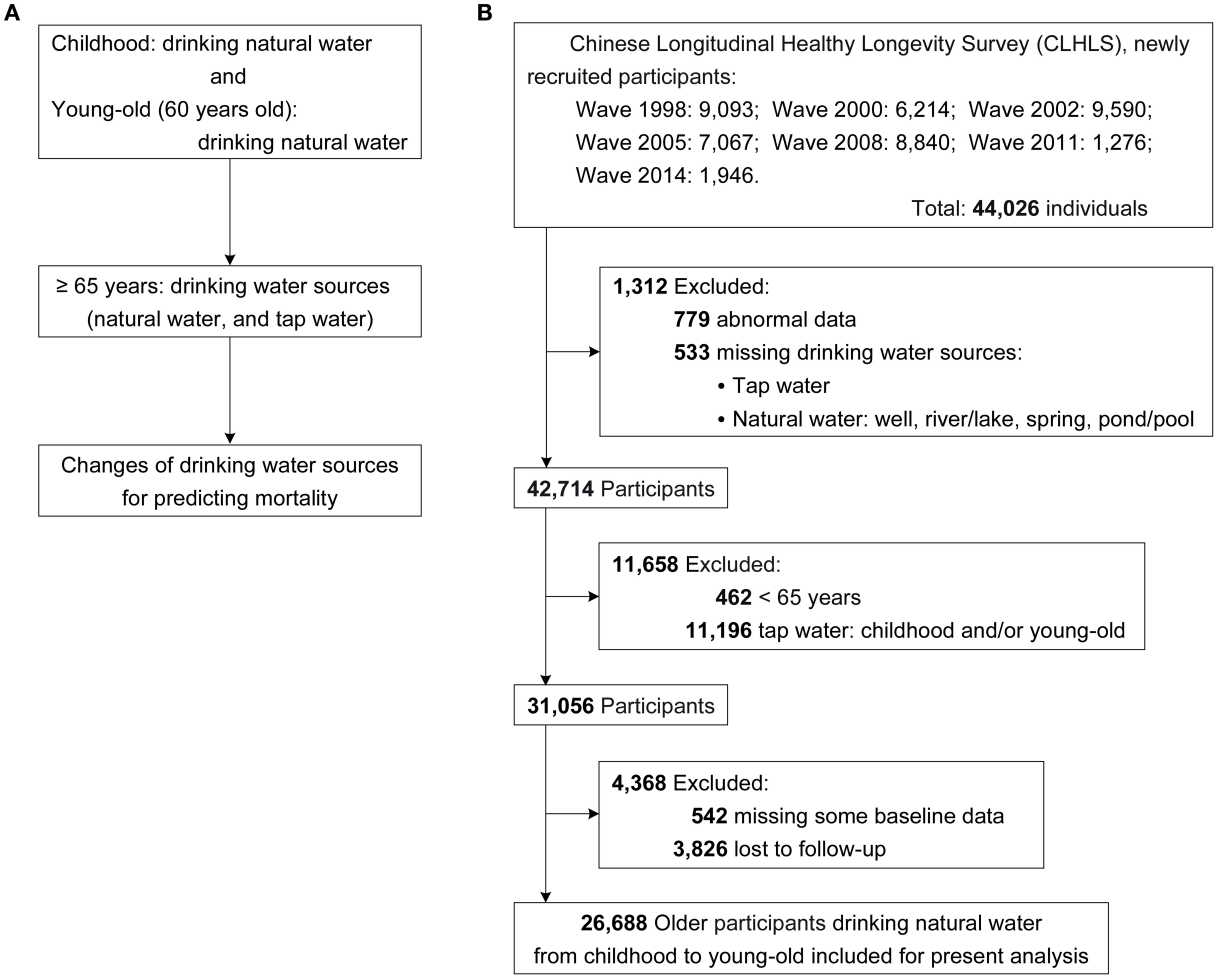
Flow chart. **(A)** study design, **(B)** participants selection diagram.

**Figure 2:**
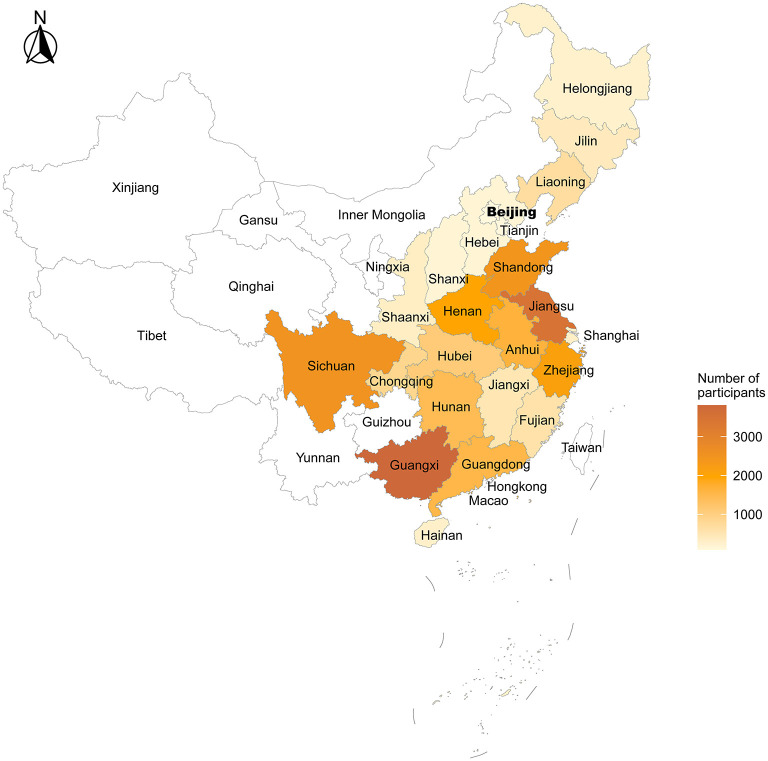
Spatial distributions of the study population. In the present study, province with the most study participants was Guangxi (*n* = 3,808), followed by Jiangsu, Sichuan, Shandong, Zhejiang, Henan, Anhui, Guangdong, Hunan, Hubei, Chongqing, Liaoning, Fujian, Jiangxi, Jilin, Shaanxi, Shanghai, Hainan, Helongjiang, Hebei, Shanxi, Tianjin, and Beijing.

The present study was based on seven waves (1998, 2000, 2002, 2005, 2008, 2011, and 2014 waves) within the CLHLS, and the final wave of interview was 2018–2019. Figure 1A shows the design of the present study, based on which the participant enrollment process was conducted (Figure 1B), and the final sample consisted of 26,688 elderly participants (age ≥ 65 years). Figure 2 shows the spatial distributions of the study population.

### Assessment of drinking water sources

At baseline, participants' drinking water sources were assessed by the questionnaire with the questions: “Water you drank at childhood was mainly from?”, “Water you drank at around 60 years old was mainly from?”, and “Water you drink at present is from?”, and the drinking water sources included well, river/lake, spring, pond/pool, and tap water. In the present study, a complex composition of drinking water sources, including wells, river/lake, spring, and pond/pool, was defined as natural water; then, the drinking water sources were classified into natural water and tap water. If the participants drank natural water at childhood and around 60 years old (young-old), they were considered to drink natural water from childhood to young-old. Using the information on drinking water sources, we compared the changes of drinking water sources, drinking natural water unchangeably and switching to tap water in later life, for association with the risk of all-cause mortality.

### Covariates

In our analyses, we also examined as many factors as possible that might be associated with drinking water sources and mortality, including sex, age, education, marital status, residence, co-residence, fresh fruits, fresh vegetables, taking meat, reading books/newspapers, current smoking, current drinking, current regular exercise, hypertension, diabetes, heart diseases, cerebrovascular diseases, respiratory diseases, cancer, self-rated health, and places of birth. Supplementary Table S1 displays the detailed information about the scales of reclassifications of baseline variables used in the present study.

### Study outcome

The study outcome was set as all-cause mortality, and the participants' survival status and date of death were collected through interviews with close family members during each survey. All individuals were followed from the first interview up to the outcome or the most recent interview.

### Statistical analysis

The missing values for all the baseline variables were no more than 0.59%, and Supplementary Table S2 shows the distributions of variables with missing data. Due to such low missing rates, the cases with missing values were deleted in the statistical analyses without imputing.

Baseline characteristics of the study population were displayed based on baseline drinking water sources (natural water vs. tap water), and the characteristics were described as median (interquartile range, IQR) for continuous variables and number (percentage) for categorical variables.

The balance in covariates was assessed by using the absolute standardized mean difference (ASD) approach, and factors with imbalance between the two groups was defined as an ASD ≥ 0.100 (19). Given the observational nature of the present study, propensity scores were developed to account for potential confounding by observed baseline characteristics (20). Propensity score methods replace an entire set of baseline characteristics with a single composite score, and this can be accomplished with a number of potential confounders in excess of what is possible with conventional regression methods. Individual propensities of participants drinking natural water were estimated with the multivariable logistic regression model that included the same covariates as the Cox regression model.

Associations between whether changing drinking water sources or not and all-cause mortality risk were then estimated by Cox regression models with the use of multiple propensity score methods. The primary analysis used propensity score matching (PSM), and 1:1 matching without replacement was performed using the nearest neighbor matching algorithm, with a fixed caliper width of 0.1. In addition, stabilized inverse probability treatment weighting (IPTW) (21) and overlap weighting (22) were performed to confirm the reproducibility of the results by PSM.

Covariate differences after PSM, as well as IPTW and overlap weighting, were assessed using the overall propensity score distributional curves and calculating the ASD for each covariate. Then, Kaplan Meier curves and Cox models that used the above-mentioned propensity score methods were reported, and we also showed the Cox model that included propensity scores as an additional covariate. To eliminate the risk of insufficient covariate balance, we also repeated the analyses by further adjusting for baseline covariates in the propensity score weighting-adjusted Cox regression models (called the “doubly robust” method) (23). Our test ascertained that the proportional hazard assumption was not been violated.

To explore the potential for unmeasured confounding between drinking water sources and risk of all-cause mortality by calculating E-values, which quantify the required magnitude of an unmeasured confounder that could negate the observed association between drinking water sources and all-cause mortality risk (24).

The statistical analyses were performed with the use of R software, version 4.1.0 (R Project for Statistical Computing), including the “survival”, “tidyverse”, “rms”, “tableone”, “survey”, “survminer”, “stats”, “MatchIt”, “cobalt”, and “RISCA” packages. For all statistical analyses, a two-sided *p*-value of 0.050 was considered statistically significant.

## Results

### Baseline characteristics

From the seven waves, the number of newly recruited participants was 44,026, and 42,714 of them completed the drinking water sources assessment, which was the primary exposure of the study. Then, 11,658 participants were excluded for not meeting the study design and hypothesis that they were under 65 at baseline or drinking tap water at their childhood and/or young-old. Of 31,056 participants, 4,368 were excluded because of the absence of baseline or follow-up data (Figure 1B). Overall, 26,688 elderly participants drinking natural water from childhood to young-old (around 60 years old) were finally included in the study. The age of the study population was 91.00 (82.00, 100.00) years presented as median (IQR) and 89.81 ± 10.76 years as mean (±SD), and 16,254 (60.90%) of the participants were women.

**Figure 3:**
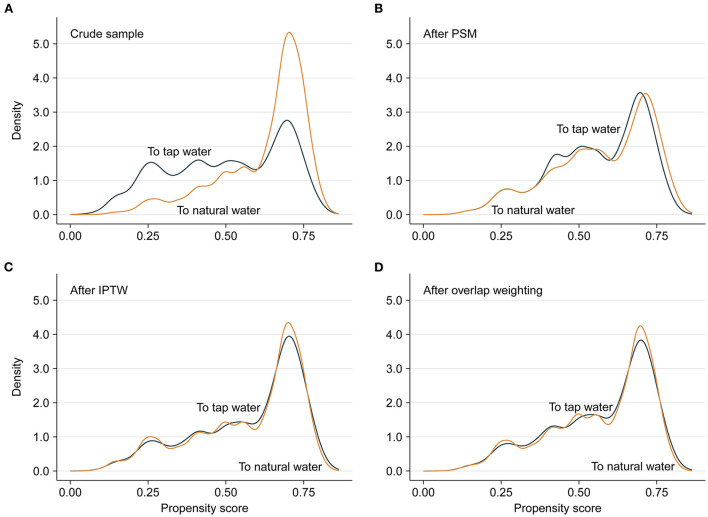
Propensity score distributional overlap before and after matching or weighting. **(A–D)** present propensity score distributions between the participants who drank natural water unchangeably and the participants who switched to tap water in the crude sample, PSM sample, IPTW sample and overlap weighting sample. For intervals along the x-axis, the area under the probability density curve represents the probability of those propensity scores, and smoothing was *via* the kernel density estimate. Greater overlap of propensity score curves of the two groups indicates a lesser risk of confounding.

In the crude sample, there was an imbalance (ASD ≥ 0.100) between the two groups in seven baseline variables, including residence, co-residence, fresh fruits, taking meat, reading books/newspapers, self-rated health, and places of birth (Table 1; Supplementary Table S3; Supplementary Figure S1). After PSM, IPTW and overlap weighting, the distributions of baseline characteristics were fairly well balanced (Figure 3); the differences were within the margin of 0.100 for all variables (Table 2; Supplementary Table S4; Supplementary Figure S1). Results of multivariable logistic regression analysis that predicted drinking natural water are listed in Supplementary Table S5, and the C-index of the propensity-score model was 0.695.

**Table 1:** Baseline characteristics stratified by baseline drinking water sources.

**Variable**	**Crude sample**		
	**To tap water (*n =* 11,421)**	**To natural water (*n =* 15,267)**	**ASD**
Sex: female	6,989 (61.2)	9,265 (60.7)	0.010
Age (years)	91.00 (83.00, 100.00)	91.00 (82.00, 100.00)	0.043
Education: 1 year or more	3,410 (29.9)	4,039 (26.5)	0.076
Marital status: not in marriage	8,759 (76.7)	11,546 (75.6)	0.025
Residence: rural	6,550 (57.4)	12,776 (83.7)	0.603
Current smoking: yes	1,871 (16.4)	2,935 (19.2)	0.074
Current drinking: yes	2,295 (20.1)	3,435 (22.5)	0.059
Current regular exercise: yes	2,705 (23.7)	3,052 (20.0)	0.089
Hypertension			0.089
No	9,239 (80.9)	12,533 (82.1)	
Yes	1,709 (15.0)	1,908 (12.5)	
Unknown	473 (4.1)	826 (5.4)	
Diabetes			0.082
No	10,764 (94.2)	14,315 (93.8)	
Yes	161 (1.4)	115 (0.8)	
Unknown	496 (4.3)	837 (5.5)	
Heart diseases			0.085
No	10,229 (89.6)	13,739 (90.0)	
Yes	723 (6.3)	727 (4.8)	
Unknown	469 (4.1)	801 (5.2)	
Cerebrovascular diseases			0.064
No	10,514 (92.1)	14,024 (91.9)	
Yes	463 (4.1)	496 (3.2)	
Unknown	444 (3.9)	747 (4.9)	
Respiratory diseases			0.058
No	9,796 (85.8)	12,900 (84.5)	
Yes	1,227 (10.7)	1,663 (10.9)	
Unknown	398 (3.5)	704 (4.6)	
Cancer			0.079
No	10,869 (95.2)	14,300 (93.7)	
Yes	42 (0.4)	33 (0.2)	
Unknown	510 (4.5)	934 (6.1)	

*Values are median (IQR) or n (%)*.

*ASD, absolute standardized mean differences*.

*Because the table is too large, some baseline information was included in the Supplementary Table S3*.

**Table 2:** Baseline characteristics stratified by baseline drinking water sources after PSM, IPTW, and overlap weighing.

**Variable**	**After PSM**			**After IPTW**			**After overlap weighting**		
	**To tap water**	**To natural water**	**ASD**	**To tap water**	**To natural water**	**ASD**	**To tap water**	**To natural water**	**ASD**
	**(*n =* 9,124)**	**(*n =* 9,124)**		**(*n =* 11,422)**	**(*n =* 15,282.4)**		**(*n =* 5,738.6)**	**(*n =* 5,738.6)**	
Sex: female	5,597 (61.3)	5,314 (58.2)	0.063	6,911.1 (60.5)	9,234.2 (60.4)	0.002	3,485.2 (60.7)	3,485.2 (60.7)	<0.001
Age (years)	91.00 (83.00, 100.00)	91.00 (81.00, 100.00)	0.079	91.00 (83.00, 100.00)	91.00 (82.00, 100.00)	<0.001	91.00 (83.00, 100.00)	91.00 (82.00, 100.00)	<0.001
Education: 1 year or more	2,551 (28.0)	2,646 (29.0)	0.023	3,220.2 (28.2)	4,306.8 (28.2)	<0.001	1,607.7 (28.0)	1,607.7 (28.0)	<0.001
Marital status: not in marriage	6,942 (76.1)	6,724 (73.7)	0.055	8,619.7 (75.5)	11,552.8 (75.6)	0.003	4,346.2 (75.7)	4,346.2 (75.7)	<0.001
Residence: rural	6,481 (71.0)	6,667 (73.1)	0.045	8,271.2 (72.4)	11,051.5 (72.3)	0.002	4,171.1 (72.7)	4,171.1 (72.7)	<0.001
Current smoking: yes	1564 (17.1)	1,784 (19.6)	0.062	2,082.8 (18.2)	2,777.1 (18.2)	0.002	1,020.2 (17.8)	1,020.2 (17.8)	<0.001
Current drinking: yes	1,909 (20.9)	2,106 (23.1)	0.052	2,470.1 (21.6)	3,286.0 (21.5)	0.003	1,221.6 (21.3)	1,221.6 (21.3)	<0.001
Current regular exercise: yes	2,009 (22.0)	2,093 (22.9)	0.022	2,481.2 (21.7)	3,328.1 (21.8)	0.001	1,242.7 (21.7)	1,242.7 (21.7)	<0.001
Hypertension			0.055			0.002			<0.001
No	7,460 (81.8)	7,327 (80.3)		9,325.1 (81.6)	12,484.4 (81.7)		4,686.4 (81.7)	4,686.4 (81.7)	
Yes	1,259 (13.8)	1,286 (14.1)		1,537.8 (13.5)	2,057.0 (13.5)		785.5 (13.7)	785.5 (13.7)	
Unknown	405 (4.4)	511 (5.6)		559.1 (4.9)	741.1 (4.8)		266.7 (4.6)	266.7 (4.6)	
Diabetes			0.051			0.005			<0.001
No	8,619 (94.5)	8,513 (93.3)		10,726.9 (93.9)	14,368.4 (94.0)		5,404.5 (94.2)	5,404.5 (94.2)	
Yes	91 (1.0)	95 (1.0)		118.3 (1.0)	153.3 (1.0)		58.4 (1.0)	58.4 (1.0)	
Unknown	414 (4.5)	516 (5.7)		576.9 (5.1)	760.8 (5.0)		275.7 (4.8)	275.7 (4.8)	
Heart diseases			0.053			0.003			<0.001
No	8,235 (90.3)	8,106 (88.8)		10,261.2 (89.8)	13,728.0 (89.8)		5,166.5 (90.0)	5,166.5 (90.0)	
Yes	490 (5.4)	521 (5.7)		610.9 (5.3)	824.3 (5.4)		309.1 (5.4)	309.1 (5.4)	
Unknown	399 (4.4)	497 (5.4)		549.9 (4.8)	730.1 (4.8)		263.1 (4.6)	263.1 (4.6)	
Cerebrovascular diseases			0.058			0.004			<0.001
No	8,421 (92.3)	8,313 (91.1)		10,498.5 (91.9)	14,048.3 (91.9)		5,286.0 (92.1)	5,286.0 (92.1)	
Yes	336 (3.7)	333 (3.6)		409.5 (3.6)	554.9 (3.6)		207.6 (3.6)	207.6 (3.6)	
Unknown	367 (4.0)	478 (5.2)		514.0 (4.5)	679.2 (4.4)		245.0 (4.3)	245.0 (4.3)	
Respiratory diseases			0.071			0.003			<0.001
No	7,838 (85.9)	7,638 (83.7)		9,697.1 (84.9)	12,989.9 (85.0)		4,897.3 (85.3)	4,897.3 (85.3)	
Yes	949 (10.4)	1,035 (11.3)		1,253.4 (11.0)	1,665.2 (10.9)		617.3 (10.8)	617.3 (10.8)	
Unknown	337 (3.7)	451 (4.9)		471.6 (4.1)	627.3 (4.1)		224.0 (3.9)	224.0 (3.9)	
Cancer			0.071			0.002			<0.001
No	8,662 (94.9)	8,515 (93.3)		10,772.3 (94.3)	14,417.6 (94.3)		5,432.0 (94.7)	5,432.0 (94.7)	
Yes	28 (0.3)	27 (0.3)		32.6 (0.3)	42.6 (0.3)		16.7 (0.3)	16.7 (0.3)	
Unknown	434 (4.8)	582 (6.4)		617.1 (5.4)	822.2 (5.4)		289.9 (5.1)	289.9 (5.1)	

*Values are median (IQR) or n (%)*.

*ASD, absolute standardized mean differences; IPTW, inverse probability treatment weighting; PSM, propensity score matching.*

*Because the table is too large, some baseline information was included in the Supplementary Table S4*.

### Association between drinking water sources and all-cause mortality

**Figure 4:**
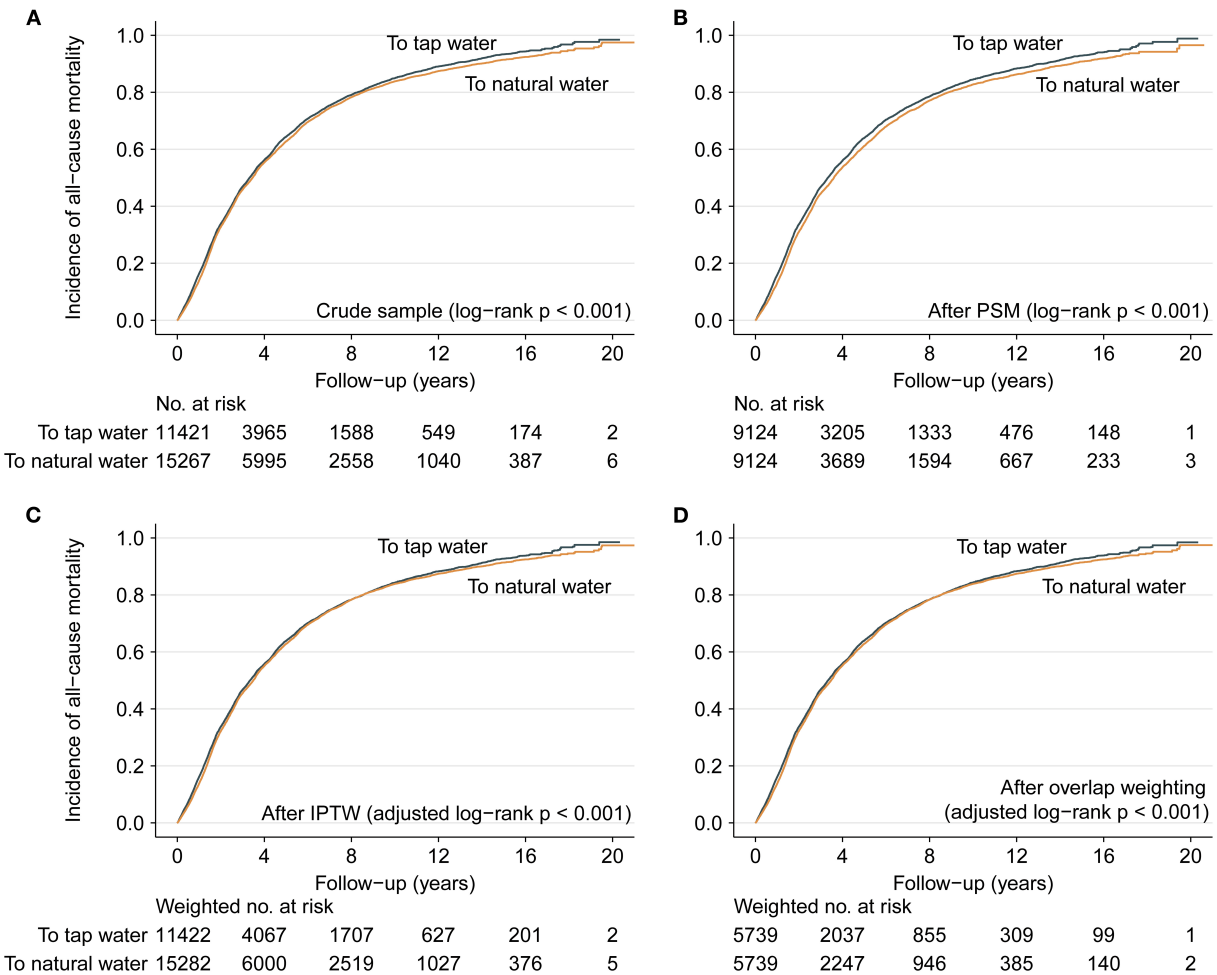
Cumulative incidence of all-cause mortality. **(A–D)** present Kaplan-Meier curves showing the participants with continued use of natural water had significantly lower cumulative incidence of all-cause mortality during the follow up in comparison with those switching to tap water later in the crude sample, PSM sample, IPTW sample and overlap weighting sample. In addition, the maximal difference of cumulative incidence was only 0.16% for the curves of “to tap water” between IPTW sample and overlap weighting sample at each same point of follow-up time, and the maximal difference was only 0.20% for the curves of “to natural water”; therefore, the curves were similar between **(C,D)**.

During a median follow-up period of 3.00 (IQR: 1.52, 5.73; Max: 21.06) years, 21,379 deaths were recorded, 8,945 of them were from the group switching to tap water and the remaining 12,434 were from the other group. Kaplan-Meier curves demonstrate that participants who drank natural water unchangeably had a lower cumulative incidence of all-cause mortality either before or after matching and weighting (all log-rank *p* < 0.001, Figures 4A–D). In the crude and multivariable analyses, participants who drank natural water unchangeably had a significantly lower all-cause mortality risk than those who switched to tap water later (**Table 3**). The primary multivariable Cox analyses with PSM yielded similar results (HR: 0.94, 95% CI: 0.91–0.97, *p* < 0.001). Results remained consistent in other additional multivariable propensity-score analyses (Table 3).

**Table 3:** Associations between drinking natural water and all-cause mortality (natural water vs. tap water).

**Analysis**	**HR (95% CI), *p***
**No. of deaths/no. of participants at risk (%)** ^**a**^
To tap water	8,945/11,421 (98.5%)
To natural water	12,434/15,267 (97.5%)
Crude analysis	0.95 (0.92–0.98), <0.001
Multivariable analysis^b^	0.94 (0.91–0.97), <0.001
**Propensity-score analyses**
With PSM (univariable)	0.92 (0.89–0.95), <0.001
With PSM (multivariable)^b^	0.94 (0.91–0.97), <0.001
With IPTW (univariable)	0.96 (0.94–0.99), 0.013
With IPTW (multivariable)^b^	0.94 (0.91–0.97), <0.001
With overlap weighting (univariable)	0.96 (0.94–0.99), 0.011
With overlap weighting (multivariable)^b^	0.94 (0.91–0.97), <0.001
Adjusted for propensity score (univariable)^c^	0.96 (0.93–0.99), 0.008
Adjusted for propensity score (multivariable)^d^	0.94 (0.91–0.97), <0.001

*Values are n (%) or HR (95% CI) with p-value*.

*^a^Cumulative incidence of all-cause mortality.*

*^b^Adjustment for sex, age, education, marital status, residence, co-residence, fresh fruits, fresh vegetables, taking meat, reading books/newspapers, current smoking, current drinking, current regular exercise, hypertension, diabetes, heart diseases, cerebrovascular diseases, respiratory diseases, cancer, self-rated health, and places of birth.*

^c^Only adjustment for propensity score.

^d^Adjustment for propensity score, and sex, age, education, marital status, residence, co-residence, fresh fruits, fresh vegetables, taking meat, reading books/newspapers, current smoking, current drinking, current regular exercise, hypertension, diabetes, heart diseases, cerebrovascular diseases, respiratory diseases, cancer, self-rated health, and places of birth.

*CI, confidence interval; HR, hazard ratio; IPTW, inverse probability treatment weighting; PSM, propensity score matching*.

Moreover, the *E*-values for the point estimate for all-cause mortality ranged from 1.20 to 1.31 with the upper limit ranging from 1.26 to 1.39 in different models (Supplementary Table S6), which might mean the robustness of the primary findings. Because in the case of the present sufficient covariates we adjusted, unless an unmeasured confounder existed with a substantially greater effect on risk of all-cause mortality (an HR higher than the *E*-values), the observed association or its CI would be reduced to null.

## Discussion

To our best knowledge, the present study was the first to investigate the relationship between different drinking water sources and all-cause mortality. Based on a prospective cohort of community-dwelling Chinese elderly people, for the participants depending on natural water for drinking from their childhood to young-old, those who drank natural water unchangeably had a significantly lower all-cause mortality risk than those who switched to tap water in later life.

People often have conflicting attitudes toward tap water (4, 7) even though it is subject to strict safety regulations and very regular inspections (25). In fact, the production of drinking water complying with international quality standards does not necessarily ensure healthy for the consumer. For instance, several factors such as concentration of organic compounds, chlorine concentration, the residence time of the water in the distribution system, water temperature, physicochemical characteristics of the material lining the distribution pipes, and detachment, accumulation, and resuspension of loose deposits can influence the regrowth of heterotrophic bacteria or the water quality in the drinking water supply system (26–28).

Owing to the high efficiency in inactivating microbial pathogens and reducing microbial growth, Chlorine is used worldwide in drinking water treatment, but meanwhile, as a strong oxidant, it causes the formation of chlorination by-products, which have been confirmed genotoxic, cytotoxic, and carcinogenic (29, 30). More than this, in comparison with bacteria isolated from an unchlorinated drinking water distribution system, chlorine-tolerant microorganisms including Legionella, Escherichia, and Geobacter in chlorinated water as bacteria isolated from a chlorinated system were more resistant to both combined and free forms of chlorine (31, 32).

Nowadays microplastics have been widely concerned as a new emerging pollutant, affecting human health in various aspects (33–35). Based on 38 tap water samples taken in different cities in China (36), Tong et al. found the amount of microplastics varied from 440 ± 275 particles/L with most of which smaller than 50 μm. Using micro-Raman spectroscopy, the authors identified 14 different materials, and the majority comprised of polyethylene and polypropylene, which are utilized in pipes in drinking water distribution systems or household (36).

Lead contamination in drinking water is also a public health issue, generally resulting from the water contact with leaded distribution piping and on premise plumbing (15). Early in 1986, US Congress amended the Safe Drinking Water Act to prohibit the use of leaded pipes, solder, and flux in public water systems (15). Lead contamination can contribute to neurotoxicity, memory reduction, reproductive toxicity, vitamin-D deficiency, cancer, and catastrophic damage to reading capability, cardiovascular and hematopoietic systems (37). A survey of lead concentration in the tap water of 29 buildings on the National Taiwan University campus revealed that faucet was a major lead source in at least 8 buildings (38).

Another issue tightly associated with tap water is water storage tanks or other similar secondary water supply systems (pumps, pipes, etc.,) commonly constructed in multi-floor and high-rise buildings in metropolises to provide adequate hydraulic pressure and equalize water demands (39). Due to some typical characteristics of these secondary water supply systems including long detention time, presence of active materials, sediment accumulation and water stratification in tanks, and warm temperature on account of sunlight exposure (e.g., outdoor tanks), disinfectant decay and subsequent microbial multiplication can take place, further leading to tap water quality deterioration (39). A study found that water storage system commonly made of commercial PVC, 304 stainless steel, and Portland cement (PTL 325) is a reservoir for several opportunistic pathogens such as P. aeruginosa, Legionella spp., mycobacteria, and V. vermiformis, which have become an emerging public health threat worldwide, especially for immunocompromised populations (40). Accumulating evidences have linked these opportunistic pathogens infections with drinking water systems (41, 42).

In contrast, drinking natural water can avoid the problems above to a certain extent. China is a large agricultural country and natural water is a common drinking source for the rural areas (43). Natural water can enhance biological stability and lower concentrations of micropollutants on account of the presence of certain bacterial and fungal species helping to promote biodegradation of organic and inorganic matter (44). It is true of natural water, which cannot be subjected to any type of disinfection that modifies or eliminates its biological components, as a result, natural water always contains bacteria that are primarily a natural component of it (10). Meanwhile, no evidence between human diseases and the natural bacteria found in natural water has been discovered (10). Additionally, owing to the adsorption by the soil and degradation by various microorganisms in the soil, spring water contained fewer contaminants like antibiotics (43).

Different types of natural waters are an excellent source of calcium, bicarbonate, magnesium, and other useful mineral substances (45). Old age is a stage with increased calcium requirements for the body, and drinking natural water rich in calcium helps to prevent osteoporosis, tooth loss, and insomnia (46). Magnesium is a protective factor against atherosclerosis, ischemic heart disease, arrhythmias, sudden death, and cerebrovascular diseases (45) which have been determined to relate to age. The high bioavailability of magnesium in natural water makes it one of the best sources of supply (45). In addition, sodium bicarbonate mineral water has also been associated with a significant drop in total cholesterol (6.3%), low-density lipoprotein cholesterol (10%), and glucemia (45).

Several limitations merit consideration. Firstly, the current sample included in the analyses was composed of Chinese elderly individuals, thus, the conclusion of these findings should be extrapolated with caution. Secondly, participants' drinking water sources in childhood were collected by the questionnaire at baseline, and it could result in recall bias. Thirdly, there is possibly some residual confounding, therefore, we further calculated E-values which ranged from 1.20 to 1.31 in the presence of adjustment for various covariates. Fourth, some information such as the daily drinking amount was not collected. Fifth, we didn't exclude the deaths from injury or accidents, further studies focused on all-mortality should exclude it to explore the relationships.

In conclusion, based on the data from CLHLS, in Chinese elderly people depending on natural water for drinking from childhood to young-old, using natural water unchangeably was associated with a significantly lower risk of all-cause mortality compared with those switching to tap water in later life. More studies, as well as comprehensive causal analyses, are needed to explore the association in different countries and populations. Last but not least, close attention should be paid to residents' drinking water safety and quality.

## Data availability statement

Publicly available datasets were analyzed in this study. This data can be found here: https://sites.duke.edu/centerforaging/programs/chinese-longitudinal-healthy-longevity-survey-clhls/survey-documentation/questionnaires/.

## Ethics statement

The studies involving human participants were reviewed and approved by Research Ethics Committee of Peking University (IRB00001052-13074). The patients/participants provided their written informed consent to participate in this study.

## Author contributions

LLiu, YZ, and SH designed the study protocol. LLiu and YZ performed statistical analysis, interpretation, and drafted the manuscript. HR, LLi, LZ, MZ, and LD performed the data collection. SH participated to the data cleaning. All authors read and approved the final manuscript.

## Funding

This study was supported by Sichuan Science and Technology Program, China (Grant No. 2022YFS0186) and the National Natural Science Foundation of China (Grant No. 81600299).

## Conflict of interest

The authors declare that the research was conducted in the absence of any commercial or financial relationships that could be construed as a potential conflict of interest.

## Publisher's note

All claims expressed in this article are solely those of the authors and do not necessarily represent those of their affiliated organizations, or those of the publisher, the editors and the reviewers. Any product that may be evaluated in this article, or claim that may be made by its manufacturer, is not guaranteed or endorsed by the publisher.
