# Supplementary material for: Drinking natural water unchangeably is associated with reduced all-cause mortality in elderly people: A longitudinal prospective study from China
Source: Front Public Health. 2022 Aug 22;10:981782. doi: 10.3389/fpubh.2022.981782 (PMC9441631; doi:10.3389/fpubh.2022.981782)
Supplement: Supplementary file 1 [file Table_1.PDF]

Supplementary Material

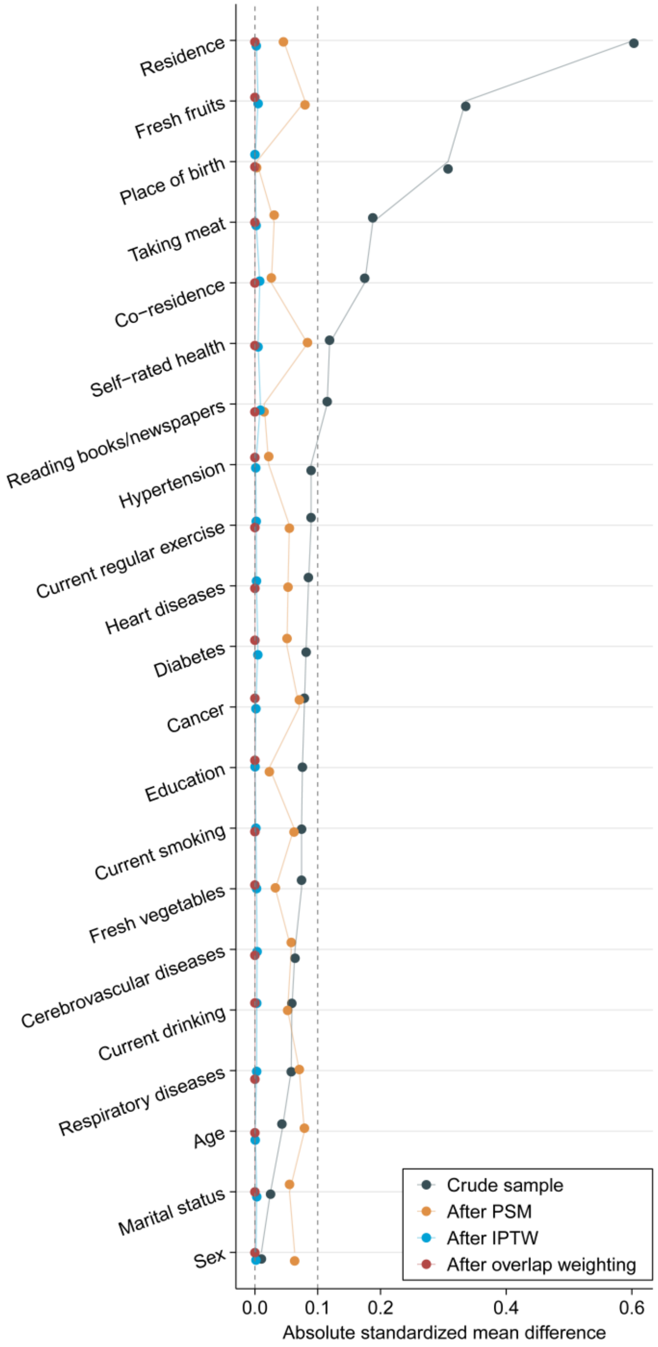

Figure S1 Absolute standardized mean differences

Absolute standardized mean differences (ASD) are shown with the participants stratified by drinking natural or tap water. ASD creates a uniform scaling by which imbalance in variables may be assessed. The dashed line indicates greater than 0.100 imbalance between the variable's values, which is a commonly used metric of significant imbalance

Table S1. Baseline variable definitions in the present study

Variable	Questions in CLHLS questionnaire	Options in the questionnaire	Scales of reclassification in the present study
Sex		• male<br>• female	• Male<br>• Female
Age	Validated age		• Age: years
Education	How many years did you attend school?	• years of school<br>• don't know<br>• missing	• No school: years of school = 0<br>• 1 year or more: years of school ≥ 1<br>• missing: don't know, missing
Marital status	Current marital status?	• currently married and living with spouse<br>• separated<br>• divorced<br>• widowed<br>• never married<br>• don't know<br>• missing	• In marriage: currently married and living with spouse, separated<br>• Not in marriage: divorced, widowed, never married<br>• missing: don't know, missing
Residence	Current residence area of interviewee?	• city<br>• town<br>• rural	• Urban: city, town<br>• Rural: rural
Co-residence	Co-residence?	• with household member(s)<br>• alone<br>• in an institution<br>• missing	• With household member(s)<br>• Alone<br>• In an institution<br>• missing

Fresh fruits	Do you eat fresh fruit?	• almost everyday<br>• quite often<br>• occasionally<br>• rarely or never<br>• don't know<br>• missing	• Often: almost everyday, except winter, quite often<br>• Occasionally: occasionally<br>• Never: rarely or never<br>• missing: don't know, missing
Fresh vegetables	Do you eat fresh vegetables?	• almost everyday<br>• quite often<br>• occasionally<br>• rarely or never<br>• don't know<br>• missing	• Often: almost everyday, except winter, quite often<br>• Occasionally: occasionally<br>• Never: rarely or never<br>• missing: don't know, missing

Taking meat	How often eat meat at present?	Waves 1998, 2000, 2002 and 2005:<br>• almost everyday<br>• occasionally<br>• rarely or never<br>• missing<br><br>Waves 2008, 2011 and 2014:<br>• almost everyday<br>• not everyday, but at least once per week<br>• not every week, but at least once per month<br>• not every month, but occasionally<br>• rarely or never<br>• don't know<br>• missing	• Often: almost everyday<br>• Occasionallys: occasionally; not everyday, but at least once per week; not every week, but at least once per month; not every month, but occasionally<br>• Never: rarely or never<br>• missing: don't know, missing

Reading books/newspapers	Do you read newspapers/books at present?	Waves 1998 and 2000:<br>• almost everyday<br>• sometimes<br>• never<br>• missing<br><br>Waves 2002, 2005, 2008, 2011 and 2014:<br>• almost everyday<br>• not daily, but once for a week<br>• not weekly, but at least once for a month<br>• not monthly, but sometimes<br>• never<br>• missing	• Almost everyday: almost everyday<br>• Sometimes: sometimes; not daily, but once for a week; not weekly, but at least once for a montlh; not monthly, but sometimes<br>• Never: never<br>• missing: missing
Current smoking	Do you smoke at the present time?	• yes<br>• no<br>• missing	• Current smoking: yes<br>• No smoking at present: no<br>• missing
Current drinking	Do you drink alcohol at the present time?	• yes<br>• no<br>• missing	• Current drinking: yes<br>• No drinking at present: no<br>• missing
Current regular exercise	Do you do exercises regularly at present?	• yes<br>• no<br>• missing	• Current regular exercise: yes<br>• No regular exercise at present: no<br>• missing

Hypertension	Are you suffering from this disease?	• yes<br>• no<br>• don't know<br>• missing	• Yes: yes<br>• No: no<br>• Unknown: don`t know<br>• missing
Diabetes	Are you suffering from this disease?	• yes<br>• no<br>• don't know<br>• missing	• Yes: yes<br>• No: no<br>• Unknown: don`t know<br>• missing
Heart diseases	Are you suffering from this disease?	• yes<br>• no<br>• don't know<br>• missing	• Yes: yes<br>• No: no<br>• Unknown: don`t know<br>• missing
Cerebrovascular diseases	Are you suffering from this disease?	• yes<br>• no<br>• don't know<br>• missing	• Yes: yes<br>• No: no<br>• Unknown: don`t know<br>• missing
Respiratory diseases	Are you suffering from this disease?	• yes<br>• no<br>• don't know<br>• missing	• Yes: yes<br>• No: no<br>• Unknown: don`t know<br>• missing
Cancer	Are you suffering from this disease?	• yes<br>• no<br>• don't know<br>• missing	• Yes: yes<br>• No: no<br>• Unknown: don`t know<br>• missing

Self-rated health	How do you rate your health at present?	• very good<br>• good<br>• so so<br>• bad<br>• very bad<br>• not able to answer<br>• missing	• Good: very good, good<br>• Fair: so so<br>• Poor: bad, very bad<br>• Unknown: not able to answer<br>• missing
Drinking water sources at childhood	Water you drank at childhood was mainly from?	• from a well<br>• from a river or lake<br>• from a spring<br>• from a pond or pool<br>• tap water<br>• don't know<br>• missing	• Natural water: well, river/lake, spring, pond/pool<br>• Tap water<br>• missing: don't know, missing
Drinking water sources around age 60 years	Water you drank at around age 60 was mainly from?	• from a well<br>• from a river or lake<br>• from a spring<br>• from a pond or pool<br>• tap water<br>• don't know<br>• missing	• Natural water: well, river/lake, spring, pond/pool<br>• Tap water<br>• missing: don't know, missing
Drinking water sources at present	Water you drink at present is from?	• from a well<br>• from a river or lake<br>• from a spring<br>• from a pond or pool<br>• tap water<br>• don't know<br>• missing	• Natural water: well, river/lake, spring, pond/pool<br>• Tap water<br>• missing: don't know, missing

Place of birth	Was the place of birth an urban area or a rural area at time of birth	Waves 1998, 2000, 2002 and 2005:<br>• urban<br>• rural<br>• missing<br><br>Waves 2008, 2011 and 2014:<br>• urban<br>• rural<br>• don't know<br>• missing	• Urban: urban<br>• Rural: rural<br>• missing: don't know, missing

Abbreviations: CLHLS = Chinese Longitudinal Healthy Longevity Surveys

Table S2. Distributions of baseline variables with missing data

Variable	Percentage with missing data[a] (%)
Sex	0.00
Age	0.00
Education	0.45
Marital status	0.06
Residence	0.00
Co-residence	0.06
Fresh fruits	0.05
Fresh vegetables	0.07
Taking meat	0.18
Reading books/newspapers	0.03
Current smoking	0.03
Current drinking	0.06
Current regular exercise	0.14
Hypertension	0.51
Diabetes	0.54
Heart diseases	0.51
Cerebrovascular diseases	0.53
Respiratory diseases	0.51
Cancer	0.59
Self-rated health	0.04
Places of birth	0.12

[a] The missing values for all the baseline variables analyzed in this research were no more than 0.59%. Due to such low missing rates, we did not impute the missing values, and we deleted the cases with missing values in the statistical analyses.

Table S3. Baseline characteristics stratified by baseline drinking water sources (Table 1 continued)

Variable	Crude sample		
	To tap water (n = 11421)	To natural water (n = 15267)	ASD
Co-residence			0.175
With family members	9472 (82.9)	12903 (84.5)	
Alone	1457 (12.8)	2145 (14.0)	
In an institution	492 (4.3)	219 (1.4)	
Fresh fruits			0.336
Never	2720 (23.8)	5132 (33.6)	
Occasionally	5100 (44.7)	7378 (48.3)	
Often	3601 (31.5)	2757 (18.1)	
Fresh vegetables			0.074
Never	416 (3.6)	579 (3.8)	
Occasionally	1427 (12.5)	2291 (15.0)	
Often	9578 (83.9)	12397 (81.2)	
Taking meat			0.188
Never	1897 (16.6)	2687 (17.6)	
Occasionally	5571 (48.8)	8590 (56.3)	
Often	3953 (34.6)	3990 (26.1)	
Reading books/newspapers			0.115
Never	9937 (87.0)	13813 (90.5)	
Sometimes	788 (6.9)	847 (5.5)	
Almost everyday	696 (6.1)	607 (4.0)	
Self-rated health			0.119
Poor	1139 (10.0)	2010 (13.2)	
Fair	3368 (29.5)	4662 (30.5)	
Good	5781 (50.6)	7004 (45.9)	
Unknown	1133 (9.9)	1591 (10.4)	
Place of birth: rural	10050 (88.0)	14686 (96.2)	0.307

Values are n (%).

Abbreviations: ASD = absolute standardized mean differences.

Table S4. Other baseline characteristics stratified by baseline drinking water sources after PSM, IPTW, and over weighing (Table 2 continued)

Variable	After PSM			After IPTW			After overlap weighting		
	To tap water (n = 9124)	To natural water (n = 9124)	ASD	To tap water (n = 11422)	To natural water (n = 15282.4)	ASD	To tap water (n = 5738.6)	To natural water (n = 5738.6)	ASD
Co-residence			0.026			0.008			<0.001
With family members	7658 (83.9)	7581 (83.1)		9557.2 (83.7)	12766.9 (83.5)		4816.8 (83.9)	4816.8 (83.9)	
Alone	1252 (13.7)	1335 (14.6)		1550.2 (13.6)	2075.3 (13.6)		778.8 (13.6)	778.8 (13.6)	
In an institution	214 (2.3)	208 (2.3)		314.5 (2.8)	440.2 (2.9)		143.0 (2.5)	143.0 (2.5)	
Fresh fruits			0.080			0.005			<0.001
Never	2455 (26.9)	2773 (30.4)		3391.6 (29.7)	4503.0 (29.5)		1621.7 (28.3)	1621.7 (28.3)	
Occasionally	4211 (46.2)	3936 (43.1)		5298.6 (46.4)	7105.5 (46.5)		2697.7 (47.0)	2697.7 (47.0)	
Often	2458 (26.9)	2415 (26.5)		2731.7 (23.9)	3673.9 (24.0)		1419.2 (24.7)	1419.2 (24.7)	
Fresh vegetables			0.033			0.003			<0.001
Never	330 (3.6)	336 (3.7)		426.5 (3.7)	575.0 (3.8)		213.8 (3.7)	213.8 (3.7)	
Occasionally	1210 (13.3)	1311 (14.4)		1556.9 (13.6)	2093.7 (13.7)		770.2 (13.4)	770.2 (13.4)	
Often	7584 (83.1)	7477 (81.9)		9438.7 (82.6)	12613.6 (82.5)		4754.6 (82.9)	4754.6 (82.9)	
Taking meat			0.031			0.002			<0.001
Never	1540 (16.9)	1554 (17.0)		1954.1 (17.1)	2603.2 (17.0)		976.3 (17.0)	976.3 (17.0)	
Occasionally	4744 (52.0)	4857 (53.2)		6043.8 (52.9)	8092.8 (53.0)		3008.8 (52.4)	3008.8 (52.4)	
Often	2840 (31.1)	2713 (29.7)		3424.1 (30.0)	4586.5 (30.0)		1753.5 (30.6)	1753.5 (30.6)	
Reading books/newspapers			0.015			0.009			<0.001
Never	8106 (88.8)	8121 (89.0)		10154.7 (88.9)	13557.1 (88.7)		5104.4 (88.9)	5104.4 (88.9)	
Sometimes	577 (6.3)	548 (6.0)		698.5 (6.1)	934.6 (6.1)		352.5 (6.1)	352.5 (6.1)	
Almost everyday	441 (4.8)	455 (5.0)		568.8 (5.0)	790.7 (5.2)		281.7 (4.9)	281.7 (4.9)	
Self-rated health			0.084			0.005			<0.001
Poor	976 (10.7)	1203 (13.2)		1354.6 (11.9)	1794.4 (11.7)		643.1 (11.2)	643.1 (11.2)	
Fair	2697 (29.6)	2744 (30.1)		3421.7 (30.0)	4574.4 (29.9)		1714.8 (29.9)	1714.8 (29.9)	
Good	4533 (49.7)	4271 (46.8)		5486.8 (48.0)	7373.0 (48.2)		2793.1 (48.7)	2793.1 (48.7)	
Unknown	918 (10.1)	906 (9.9)		1159.0 (10.1)	1540.5 (10.1)		587.5 (10.2)	587.5 (10.2)	
Place of birth: rural	8575 (94.0)	8581 (94.0)	0.003	10588.4 (92.7)	14166.6 (92.7)	<0.001	5373.1 (93.6)	5373.1 (93.6)	<0.001

Values are n (%).

Abbreviations: ASD = absolute standardized mean differences, IPTW = inverse probability treatment weighting, PSM = propensity score matching.

Table S5. Multivariable logistic regression model predicting drinking natural water

Variable	Changes	Beta coefficient	Std.error	p value
Sex	Female vs. male	0.028	0.035	0.418
Age	per 1-year increment	-0.006	0.002	<0.001
Education	1 year or more vs. No school	-0.034	0.038	0.366
Marital status	Not in marriage vs. In marriage	-0.015	0.039	0.701
Residence	Rural vs. Urban	1.165	0.031	<0.001
Co-residence				
With family members		ref.		
Alone		-0.009	0.040	0.820
In an institution		-0.726	0.088	<0.001
Fresh fruits				
Never		ref.		
Occasionally		-0.226	0.032	<0.001
Often		-0.767	0.039	<0.001
Fresh vegetables				
Never		ref.		
Occasionally		0.170	0.078	0.030
Often		0.098	0.072	0.174
Taking meat				
Never		ref.		
Occasionally		0.124	0.037	0.001
Often		-0.124	0.041	0.003
Reading books/newspapers				
Never		ref.		
Sometimes		-0.107	0.060	0.075
Almost everyday		-0.062	0.068	0.360
Current smoking: yes	yes vs. no	0.149	0.038	<0.001
Current drinking: yes	yes vs. no	0.088	0.034	0.010
Current regular exercise: yes	yes vs. no	0.048	0.034	0.151
Hypertension				
No		ref.		
Yes		-0.188	0.040	<0.001
Unknown		0.071	0.104	0.496
Diabetes				
No		ref.		
Yes		-0.478	0.133	<0.001
Unknown		-0.082	0.135	0.540
Heart diseases				
No		ref.		
Yes		-0.199	0.060	0.001
Unknown		-0.054	0.139	0.696
Cerebrovascular diseases				
No		ref.		
Yes		-0.198	0.072	0.006

Unknown		-0.146	0.152	0.337
Respiratory diseases				
No		ref.		
Yes		0.010	0.044	0.817
Unknown		0.186	0.137	0.174
Cancer				
No		ref.		
Yes		-0.643	0.248	0.010
Unknown		0.339	0.102	0.001
Self-rated health				
Poor		ref.		
Fair		-0.218	0.047	<0.001
Good		-0.279	0.046	<0.001
Unknown		-0.212	0.059	<0.001
Place of birth	Rural vs. Urban	0.643	0.056	<0.001

Individual propensities of participants drinking natural water were estimated with the use of a multivariable logistic regression model that included all the baseline variables. C-index=0.695.

Table S6. E-values for the corresponding HRs in Table 3

Analysis	E-value[a] (upper CI)
Crude analysis	1.23 (1.31)
Multivariable analysis	1.26 (1.34)
Propensity-score analyses	
With PSM (univariable)	1.31 (1.39)
With PSM (multivariable)	1.26 (1.34)
With IPTW (univariable)	1.20 (1.26)
With IPTW (multivariable)	1.26 (1.34)
With overlap weighting (univariable)	1.20 (1.26)
With overlap weighting (multivariable)	1.26 (1.34)
Adjusted for propensity score (univariable)	1.20 (1.28)
Adjusted for propensity score (multivariable)	1.26 (1.34)

[a] Correspond to the HRs in Table 3.

Abbreviations: CI = confidence interval, HR = hazard ratio, IPTW = inverse probability treatment weighting, PSM = propensity score matching.
